# Sooty Tern Optimization Algorithm-Based Deep Learning Model for Diagnosing NSCLC Tumours

**DOI:** 10.3390/s23042147

**Published:** 2023-02-14

**Authors:** Muhammad Asim Saleem, Ngoc Thien Le, Widhyakorn Asdornwised, Surachai Chaitusaney, Ashir Javeed, Watit Benjapolakul

**Affiliations:** 1 Center of Excellence in Artificial Intelligence, Machine Learning and Smart Grid Technology, Department of Electrical Engineering, Faculty of Engineering, Chulalongkorn University, Bangkok 10330, Thailand; 2 Aging Research Center, Karolinska Institutet, 171 65 Stockholm, Sweden

**Keywords:** lung cancer, deep learning model, sooty tern optimization algorithm (STOA), non-small cell lung cancer (NSCLC)

## Abstract

Lung cancer is one of the most common causes of cancer deaths in the modern world. Screening of lung nodules is essential for early recognition to facilitate treatment that improves the rate of patient rehabilitation. An increase in accuracy during lung cancer detection is vital for sustaining the rate of patient persistence, even though several research works have been conducted in this research domain. Moreover, the classical system fails to segment cancer cells of different sizes accurately and with excellent reliability. This paper proposes a sooty tern optimization algorithm-based deep learning (DL) model for diagnosing non-small cell lung cancer (NSCLC) tumours with increased accuracy. We discuss various algorithms for diagnosing models that adopt the Otsu segmentation method to perfectly isolate the lung nodules. Then, the sooty tern optimization algorithm (SHOA) is adopted for partitioning the cancer nodules by defining the best characteristics, which aids in improving diagnostic accuracy. It further utilizes a local binary pattern (LBP) for determining appropriate feature retrieval from the lung nodules. In addition, it adopts CNN and GRU-based classifiers for identifying whether the lung nodules are malignant or non-malignant depending on the features retrieved during the diagnosing process. The experimental results of this SHOA-optimized DNN model achieved an accuracy of 98.32%, better than the baseline schemes used for comparison.

## 1. Introduction

Early lung cancer is generally present statistically in the form of a solitary pulmonary nodule. This solitary pulmonary nodule is highly complex and crucial for performing the segmentation process over a sequence of lung parenchyma images at a faster rate without compromising accuracy [1]. Thus, the segmentation method is essential for subsequent achievement of lung cancer nodule segmentation and diagnosis in order to differentiate benign and malignant features from the input CT images [2]. Specifically, lung parenchyma segmentation represents partitioning the lung parenchyma into a finite number of regions of interest with particular potential properties. In this context, computer-aided diagnosis (CAD) of pulmonary diseases opened wide the way to identifying suspected pulmonary nodules from an image-processing perspective [3]. In lung cancer, pulmonary nodules exist in the shape of an abnormal spheroidal tissue present in the complex juxtapleural structure derived from CT lung images [4]. However, the surrounding tissue of the lungs, such as blood vessels and chest walls, have the maximized probability of hindering the process of detection or segmentation during the staging and diagnosis process [5]. In terms of statistics, solitary pulmonary nodules are considered to constitute approximately 6% to 17% of juxtapleural nodules. Most segmentation methods that contributed to the literature initially concentrated on the grey-level thresholding mechanism, since these nodules, as mentioned above, are attached to the pleura, with their associated greyness and density being very similar to those of the pleura [6]. Thus, a potential automated segmentation method is essential during the segmentation of juxtapleural nodules, which often occurs in the region of depression in the lung parenchyma, generally named the juxtapleural nodular depression [7]. This automated segmentation method must be developed to handle complete types of pulmonary nodules, focusing on achieving indispensable segmentation results with a potential impact on accuracy under image analysis, auxiliary, and other post-processing functions [8].

Further, the automated segmentation process is necessary to prevent the limitations of under-segmentation and edge leakage during its employment against lung parenchyma complex structures [9]. As a result, segmentation is responsible for the potential derivation of the necessitated area of interest through detainment of the lungs’ outline details and good edges. It is also essential to segment juxtapleural and juxtavascular nodules that contribute to the effectiveness of ancillary diagnosis with repaired depressed areas [10]. The automatic segmentation approach hugely reduces an expert’s image analysing time (per patient, a CT scan contains 100s of image slices), minimizes the false-positive rate, improves segmentation accuracy, aids in achieving precise classification results, and improves 3D visualization quality. Deep learning (DL) focuses not only on the depth of the learning model but also on the prominence of feature-learning facilitated over the network model [11]. It has established its stand in the research of NSCLC detection. DL has become predominant in NSCLC detection with its heavy computational requirements. This becomes possible with the powerful graphics processing units (GPUs) available [12]. Though there are conventional methods for NSCLC detection, the methods based on DL rely less on pre-processing methods and feature-based models. This is made possible by ‘end-to-end’ learning from the input images [13]. DL is an innovation in computer vision that can be potentially applied to recognize cancerous tumours from the organs of humans such as the lungs, cervix, and breast. Initially, the regions of the organs susceptible to infection through cancer are considered input images, and the deep network is applied to identify the accurate location under consideration. Among several DL-based models, convolutional neural network (CNN) is the best and is extensively used, as it facilitates independent learning. It is used in diverse fields, including object recognition, face and emotion recognition, and cancer detection. Feature maps (FMs) are formed by passing images taken as input through filters found in the convolution layer [14]. They form a fully connected network. Based on the output of the Softmax function, an NSCLC tumour was detected with its type and stage. There are diverse DL neural networks (DLNNs) involved in the NSCLC tumour detection process. Some of the predominant deep-learning algorithms are widely applied in computer vision to determine features and classify and recognize the region of interest (RoI) in the classification process [15].

This paper proposes a sooty tern optimization algorithm-based DL model for diagnosing NSCLC tumours with increased accuracy. This diagnosing model perfectly isolates the lung nodules by adopting the Otsu segmentation method. Then, the sooty tern optimization algorithm (SHOA) is adopted for partitioning the cancer nodules by defining the best characteristics, which aids in improving diagnostic accuracy. It further utilizes a local binary pattern (LBP) for determining the relevant feature retrieval from the lung nodules. In addition, it adopts CNN- and GRU-based classifiers for identifying whether the lung nodules are malignant or non-malignant depending on the features retrieved during the diagnosing process. Experimental validation of the proposed SHOA-DNN model is achieved using accuracy, precision, recall, and the F-score under different classes of CT images.

The remaining sections of the paper are structured as shown below. Section 2 gives a comprehensive view of existing optimized DL models that have contributed to the literature for diagnosing lung cancer, with merits and limitations. Section 3 highlights the detailed view of the proposed SHOA-DNN model with suitable diagrams. Section 4 demonstrates the experimental validation of the proposed SHOA-DNN model with benchmarked schemes used for comparison. Section 5 gives the conclusion, with significant contributions and future possibilities for improvement.

## 2. Related Works

This section presents some important classification mechanisms propounded over recent years for diagnosing cancerous lung cancer nodules, with merits and limitations.

An end-to-end convolutional neural network (CNN)-based classification scheme was proposed by Masud et al. [16] for automatic lung nodule detection with minimized false-positives. This CNN architecture-based classification scheme was adopted with four different convolutional layers. Therefore, it is considered to facilitate a better classification process. The CNN architecture’s layers comprise a pooling block, non-linear activation functions after every block, a connector convolutional block, and two consecutive convolutional blocks. The experimental investigation of this CNN-based classification approach was carried out through the LIDC database that included 1279 sample images, of which 278 were benign, 568 were non-cancerous, and the remaining were malignant. It achieved an accuracy of 97.9% compared to other existing CNN-based lung nodule classification approaches. Jiang et al. [17] proposed a multigroup, patches cut-based lung nodule classification scheme to improve the accuracy of cancer diagnosis. This classification process was contributed with the merits of the Frangi filter that aided in preventing strictness in performance for the standards of the database. It specifically included four-channel CNN to attain knowledge about four levels of lung nodules through the integration of two groups of images. This computer-aided system confirms a sensitivity of 99.4% with a 15.6 false-positive rate per scan and a sensitivity of 80.06% with a 4.7 false-positive rate per scan.

A deep model-learning information (fuse-texture, shape, and deep model (TSD))-based lung nodule classification approach was proposed by Xie et al. [18] for incorporating texture and shape features at the decision level. It utilized deep CNN to automatically learn feature representations associated with lung nodules, determined in a slice-by-slice manner. It employed the Fourier shape descriptor and grey-level co-occurrence matrix (GLCM)-based texture descriptor for determining the characterization of the lung nodules’ heterogeneity. It also used individual feature types and integrated the decisions facilitated by the included classifiers toward differentiating normal and cancerous lung nodes based on AdaBoost backpropagation neural network (BPNN) training. The experimental validation of this deep-CNN model was conducted using the LIDC-IDRI dataset and considered only the lung nodes with a malignancy rate greater than 3 mm during the classification process. This Fuse-TSD algorithm was identified to achieve an AUC of 0.96, on par with the AUC attained using existing classification approaches. Then, the SVM and wavelet feature descriptor-based classification scheme was proposed by Madero Orozco et al. [19] for supervised region-of-interest extraction that prevents the differences among features derived from CT images. It utilized the benefits of Daubechies db1, db2, and db4 wavelet transforms for computing primary and secondary decomposition levels. If further computed that 19 features are associated with each wavelet sub-band once the decomposition process is completed. It also performed attribute and sub-band selection to identify and integrate 11 features that need to be given as input to SVM for differentiating between normal and cancerous lung nodules. The experimental validation of this SVM classification approach was conducted using ELCAP and LIDC datasets that comprised 105 CT images. Altogether, 61 CT images, which included 36 and 25 cancerous and normal lung nodules, respectively, were used during the training stage. The testing was performed using 45 CT scans composed of 23 cancerous and 22 typical lung nodules.

A multi-crop convolutional neural network (MCCNN)-based lung cancer nodule classification scheme was proposed by Shen et al. [20] by exploring and handling the issue of lung nodule malignancy suspiciousness that could be feasibly derived from CT images. It was proposed to specifically concentrate on handling time-consuming feature extraction and cautious nodule segmentation. It utilized MCCNN and a significant multi-crop pooling strategy to attain the automatic extraction of potent nodule information that plays an anchor role in cropping potential areas from convolutional FMs. It further implemented a max-pooling operation several times to handle the challenge inherent in the process of modelling raw nodule patches. It furthermore constructed end-to-end machine learning (L) frameworks that aid in categorizing lung nodules based on the factor of malignancy suspiciousness. The results of this MCCNN model also confirmed better sensitivity and precision of 98.21% and 98.56%, respectively, compared to the existing literature. A two-deep, three-dimensional (3D), customized, mixed-link network (CMixNet)-based DL model was proposed by Nasrullah et al. [21] for precise detection and classification of malignant lung nodules. It facilitated nodule detection using faster R-CNN and potentially-learned features based on the encoder–decoder architecture of CMixNet and U-Net. It specifically attained lung nodule classification based on the inclusion of a gradient-boosting machine (GBM) that has the probability of learning automatic features from the utilized 3D CMixNet structure. The evaluation of the CMixNet-based DL model conducted using LIDC-IDRI datasets confirmed better sensitivity and specificity of 94% and 91%, respectively, on par with the existing lung cancer diagnosis DL models.

Kirienko et al. [22] proposed a CNN-based classification scheme that categorizes T1-T2 or T3-T4 stages of lung cancer lesions that could possibly be identified from CT or fluorodeoxyglucose positron emission tomography (FDG-PET) images. This method used the seventh edition of the TNM system as a reference to perform the classification process of lung nodules. It enforced pre-processing with the objective of adequately generating the dataset. This CNN model considered the bounding box, cropped PET, and CT images as input for classification. It classified the lung nodules into concordant and discordant, depending on the deviation between the reference and prediction strategy. The feature extractor and classifier-adopted model of CNN was determined to achieve an AUC, specificity, recall, and accuracy of 0.68, 67%, 47%, and 90%, respectively, during the validation process. Xie et al. [23] proposed a multi-view, knowledge-based, collaborative (MV-KBC) deep model-based analysis of lung cancerous nodules for facilitating reliable classification between malignant and benign nodules using restricted chest CT data. This MV-KBC deep model was contributed with the characteristics of 3D lung nodules to classify them into nine potential views. It constructed a knowledge-based collaborative (KBC) sub-model to fine-tune the features based on the merits of ResNet-50 networks that segregated malignant from benign nodules. It used ResNet-50 networks for characterizing shape heterogeneity, voxel, and the overall appearance of the lung nodules toward the detection of lung cancer. It also included nine sub-models of KBC during error backpropagation and classified lung nodules based on the added advantages of adaptive weighting-scheme learning. It utilized the function of penalty loss to minimize the false negative rate, which introduced minimal impact on the complete performance of the KBC model. The results of this MV-KBC deep model proved its predominance, with an AUC and accuracy of 0.957 and 91.60%, respectively, during the lung classification process.

Lakshmanaprabu et al. [24] proposed a linear discriminate analysis (LDA) and optimal deep neural network (ODNN)-based lung nodule classification of lung images from CT images. This ODNN model extracted deep-level features from CT lung images and utilized LDA to reduce the features’ dimensionality. It was proposed for classifying malignant and benign lung nodules from CT images. It included a modified gravitational search algorithm (MGSA) for optimizing the features that the ODNN model learns to identify in the cancerous lung nodules during classification. The outcomes confirm 94.56% accuracy, 94.2% specificity, and 96.2% sensitivity compared to baseline cancer-detection models. A DCNN-integrated NoduleX-based lung nodule malignancy detection mechanism was proposed by Causey et al. [25] to predict the malignancy of lung nodules from CT data. This NoduleX-based lung nodule malignancy detection scheme explored more than 1000 lung nodules derived from images taken from the LIDC/IDRI cohort during training and validation. It was proposed with significant feature extraction and optimization, which aided in attaining maximized accuracy during nodule malignancy classification. This NoduleX scheme was a practical framework to accurately predict malignancy depending on the model trained using a large patient population. Astaraki et al. [26] proposed a dual pathway model to capture contextual information and intra-nodule heterogeneities associated with pulmonary nodules. This DPM-DNN model was proposed with the merits of both unsupervised and supervised learning strategies for gaining maximized accuracy during the classification process [27]. It adopted a random forest model as the second entity over the top of the networks with the objective of generating better classification results. This model included different powers of discrimination during the classification process and confirmed superior ROC during its investigation, conducted using 1297 manually segmented lung nodules. It also integrated target and context-supervised DL features, which aided in attaining a discrimination power of 0.936 based on existing works in the literature.

## 3. Proposed Methods

**Figure 1:**
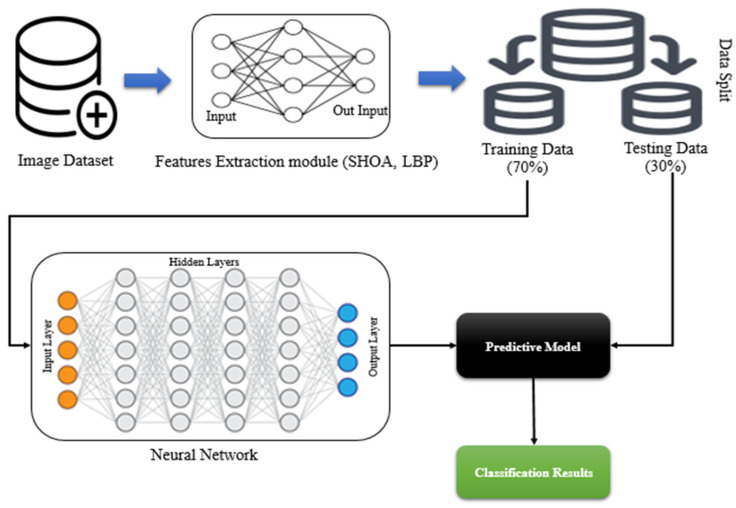
Block diagram of the proposed model.

The proposed SHOA-DNN model implemented for diagnosing lung cancer comprises four essential steps: (i) segmentation using automatic lung parenchyma mining and border restoration (ALPM & BR), (ii) optimization of features using SHOA, (iii) LBP-based optimized feature extraction, and (iv) a CNN-GRU-based classification process. The working of the proposed model is given in Figure 1.

### 3.1. Automatic Lung Parenchyma Mining and Border Restoration (ALPM & BR)

Segmentation of the lung is a predominant step in automatically investigating chest CT images. Segmentation includes isolating lungs by eliminating the tissue outside the parenchyma and faultlessly finding the edges. Nodules are seen within the LP at diverse positions, such as main and lobar bronchus, pleura, mediastinum, etc. In the identification process, if segmentation does not correctly mine the boundaries of the wall, then the nodules invaded to the edges may not be considered and may be partly or entirely missed. Thus, an automatic and precise segmentation mechanism is necessary. The detailed segmentation algorithm is shown below.

#### 3.1.1. Automatic Single-Seeded Region Growth (ASSRG) Algorithm

**Figure 2:**
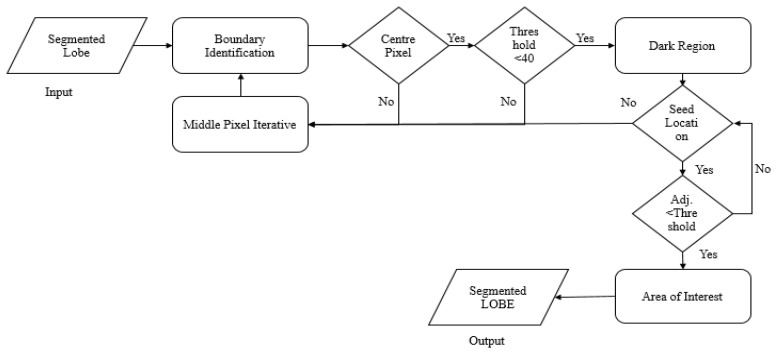
Flow chart of ASSRG algorithm.

The lobes are occupied with air, and therefore the region shows less disparity and similar contrast to the adjacent region. The region-growing segmentation (RGS) scheme divides the lobe areas using the propounded single-seed selection procedure. Based on the intensity, shape, and location, a single seed choice is propounded, and region mining is performed as detailed in Algorithm 1. Figure 2 provides the information for Algorithm 1.
**Algorithm 1:** ASSRG **Input:** ‘IL’— Either right or left lung region **Output:** Lobe that is segmentedChoose the single seed pixel location and image intensity.
Give ‘IL’ as input.Determine the central pixel.Determine whether the region with an intensity threshold less than 40 repre-sents air in the lung.Find whether the high number of neighbours are dark regions.In maximum cases of dark regions, take that pixel as the seed location and intensity.Otherwise, develop the middle pixel iteratively in all directions.Redo steps (c), (d), and (e).
Determine adjacent pixels based on the difference found between the adjoining pixels and the seed intensity if it is less than the threshold. Add that adjacent position to ex-tract the region of interest (lung lobe).Develop seed areas by incorporating neighbouring pixels that fulfil the comparison rule.Redo step 2 intended for every fresh pixel; halt if no neighbouring pixels are involved with the region of interest.Output the segmented lung lobe (right or left) region.The algorithm divides the lobe with a precise edge for remote nodules. For juxtavas-cular or juxtapleural nodules, it produces a lobe edge with concavity.

#### 3.1.2. Novel Hybrid Border Concavity Closing (NHBCC) Algorithm

**Figure 3:**
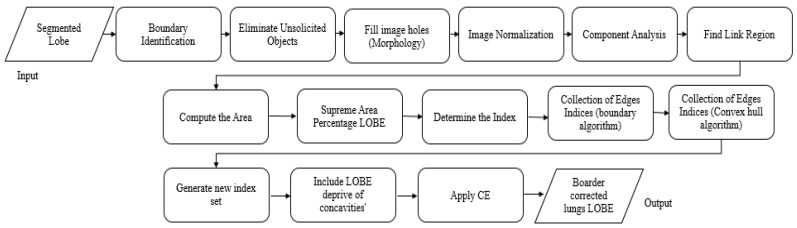
Flow chart of NHBCC algorithm.

ASSRG is a lobe area that may include concavity on the edge due to surrounding organs and anatomic structures and the presence of juxtavascular and juxtapleural nodules. Eliminating boundary concavity may lead to extracting the perfect lung lobe, which helps to isolate nodules invaded by lung walls. Hence, the NHBCC scheme is propounded as detailed in Algorithm 2. The propounded scheme uses the morphological function, related component investigation, and logical rules to mine lobe areas depending on the area. Hybrid boundary identification and a convex hull algorithm are applied to gather boundary indices by eliminating minor and major deep-concave indices using a clockwise line linking the gathered edge indices with the width of the line (n ≥ 4), which generates a thick line edge deprived of concavity. The canny edge (CE) identification is used to identify dense-edge lines’ internal and external edges. To circumvent highly-segmented lung walls, the internal boundary of a thick line is mined by computing the area that encircles the whole lung lobe area. The propounded scheme guarantees the complete separation of juxtavascular and juxtapleural nodules from the outside wall or mediastinum area. Figure 3 provides the overview of the NHBCC algorithm.
**Algorithm 2:** NHBCC Algorithm **Input:** ASSRG segmented lobe (right or left) (J), the width of the line (n) **Output**: Border-corrected lung lobe (Ibcl)Add ‘n’ columns of zeros to the ‘J’ at the left and right sides to enhance the efficacy of boundary identification.Apply a morphological opening function to eliminate unsolicited objects from the image.Morphologically rebuild based on the flood-fill function to fill the holes in the images.Normalize the shape of the image using MF.Implement associated component analysis.Determine the number of linked regions (N).for j = 1:NCompute the area (j) of every associated regionendMine the associated region (Iar) that has the supreme area % of lobe area.Determine the indices of ‘Iar’ that are genuinely intense and save them as φ=(x(i), y(i)), where ‘i’ is the number of locations with true intensity.Implement the revealing boundary algorithm with ‘φ’ indexes set, which produces a collection of edge indices (χ). % negates minor deep concave indices.Implement a convex hull algorithm with ‘φ’ indexes set that produce a collection of edge indices (£). % negates massive deep concave indices.Generate new index set ‘s’ by linking ‘χ’ and ‘£’ indices (if identical indices, preserve a duplicate).Link indices (s) with line width ‘n’ that wholly includes a lobe deprived of concavities.Apply CE to the outcome of step 10 and detect boundaries.Redo steps 5 to 7.Mine the linked area that has the smallest area (I_min). % (Inner lobe boundary deprived of highly segmented external soft wall)The internal area of ‘I_min’ is made intense. Eliminate the intentionally added columns of zeros in step 1.Return the border-corrected lobe region (Ibcl).

### 3.2. Optimization of Features Using the SHOA Algorithm

**Figure 4:**
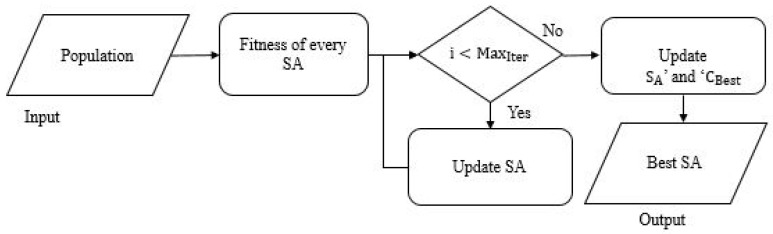
Flow chart of STOA algorithm.

The process of feature optimization achieved using inspiration derived from the sooty tern optimization algorithm (SHOA) is detailed below [28]. It is mainly adopted for partitioning the nodules of cancer by defining the best characteristics, which aids in improving diagnostic accuracy. The implementation of SHOA towards feature optimization is achieved using the phases of migration and attack, representing exploration and exploitation, respectively. Algorithm 3 provides the working of SHOA for feature optimization, and Figure 4 provides the flow chart of STOA algorithm.

#### 3.2.1. Migration (Exploration)

While migrating, an ST should fulfil the ensuing conditions.

- Collision evasion: ‘$M_{\mathrm{SA}}$’ gives the new position of a search agent (SA) that deals with avoiding collisions amid the adjacent SAs (STs).

(1) $${\vec{C}}_{\mathrm{ST}}^{L}=M_{\mathrm{SA}}\times {\vec{P}}_{\mathrm{ST}}^{L}$$

where

${\vec{C}}_{\mathrm{ST}}^{L}$—Location of SA that does not affect that of other SAs;
${\vec{P}}_{\mathrm{ST}}^{L}$—Present location of SA;
$M_{\mathrm{SA}}$—Movement of SA in assumed search space.

(2) $$M_{\mathrm{SA}}=C_{\mathrm{fac}}-\left(i\times \frac{C_{\mathrm{fac}}}{{\mathrm{Max}}_{\mathrm{Iter}}}\right)$$

where

i —Present iteration, $i\;=0,\;1,\;2,\ldots .{\;\mathrm{Max}}_{\mathrm{Iter}}$;
$C_{\mathrm{fac}}$—Controlling factor (set to 2), which modifies ‘$M_{\mathrm{SA}}$’ linearly decreased to 0.

- Converge in the direction of the best neighbour: once a collision is overcome, SAs converge in the track of the best neighbour.

(3) $${\vec{M}}_{\mathrm{ST}}^{L}=C_{\mathrm{Best}}\times \left({\vec{P}}_{\mathrm{BST}}^{L}\left(i\right)-{\vec{P}}_{\mathrm{ST}}^{L}\left(i\right)\right)$$

where

${\vec{M}}_{\mathrm{ST}}^{L}$—Diverse locations of SA $\left({\vec{P}}_{\mathrm{ST}}^{L}\right)$ towards the best, fittest SA $\left({\vec{P}}_{\mathrm{BST}}^{L}\right)$;
$C_{\mathrm{Best}}$—Random variable employed for improved exploration.

(4) $$C_{\mathrm{Best}}=0.5\times \mathrm{Ran}$$

where

Ran —Random number that is in the range [0, 1].

- Updation conforming best SA: lastly, SA or ST modifies its location based on the best SA.

(5) $${\vec{G}}_{\mathrm{ST}}^{L}={\vec{C}}_{\mathrm{ST}}^{L}+{\vec{M}}_{\mathrm{ST}}^{L}$$

where

${\vec{G}}_{\mathrm{ST}}^{L}$—Gap amid the SA and fittest SA.

#### 3.2.2. Attacking (Exploitation)

While migrating, STs modify their velocity along with the attacking angle. Wings are employed to raise their altitude [29]. They fly in spirals when attacking their prey.

(6) $$X^{\prime }=\mathrm{Rad}\;\times \mathrm{Sin}\left(a\right)$$

(7) $$Y^{\prime }=\mathrm{Rad}\;\times \mathrm{Cos}\left(a\right)$$

(8) $$Z^{\prime }=\mathrm{Rad}\;\times a$$

(9) $$r=u\;\times e^{\mathrm{kv}}\;$$

where

Rad—Radius of every spiral turn;

A—Range of [0≤k≤2π];

u, v—Constants representing spiral, assumed to be ‘1′;

e—Natural algorithm’s base.

The modified location of SA is obtained using Equations (8)–(10).

(10) $${\vec{P}}_{\mathrm{ST}}^{L}\left(i\right)=\left({\vec{G}}_{\mathrm{ST}}^{L}\times \left(\;X^{\prime }+\;Y^{\prime }+\;Z^{\prime }\right)\right)\times {\vec{P}}_{\mathrm{BST}}^{L}\left(i\right)$$

where

${\vec{P}}_{\mathrm{ST}}^{L}\left(i\right)$—Modifies locations of other SAs and keeps the best ideal solution.
**Algorithm 3:** STOA**Input:**$\;\mathrm{Population}\;\left({\vec{P}}_{\mathrm{ST}}^{L}\left(i\right)\right)$**Output:**$\;\mathrm{Best}\;\mathrm{SA}\;\left({\vec{P}}_{\mathrm{BST}}^{L}\left(i\right)\right)$$\mathrm{Initialize}\;‘M_{\mathrm{SA}}$$’\;\mathrm{and}\;‘C_{B}$’  Determine the fitness of every SA    $\mathrm{while}\;(i<{\mathrm{Max}}_{\mathrm{Iter}}$) do      for every SA, do      Modify the locations of SAs using Equation (10)    end //for  $\mathrm{Update}\;‘S_{A}$$’\;\mathrm{and}\;‘C_{\mathrm{Best}}$’  Find the fitness of every SA$\mathrm{Modify}\;‘{\vec{P}}_{\mathrm{BST}}^{L}\left(i\right)$’ in case there is an improved solution compared to a former ideal solutioni=i+1$\mathrm{return}\left({\vec{P}}_{\mathrm{BST}}^{L}\left(i\right)\right)$End

#### 3.2.3. Improved LBP-Based Optimized Feature Extraction

In the proposed SHOA-DNN model, an improved LBP-based feature extraction method is adopted using the merits of an adaptive threshold and representation of directional local image features. It is adopted explicitly to realize the process of image retrieval. This process of local integration comprises two parts that include (i) determining the standard deviation between the grey value of all the pixels in the CT image’s local neighbourhood and the grey value of the pixel present in the centre used for encoding, and (ii) determining the local neighbourhood pixels’ grayscale changes concerning different binary encoding, discrimination, and direction. Finally, the optimized features are considered for image retrieval by combining local features inherent to the image. It aided in the detection of automatic seed-point identification, region growing-based mining, and new border concavity closing methods to attain impeccable mining that aids in isolating lungs by excluding the surrounding area. The nodules are mined using a CCA and TBMN improvement process that efficiently eradicates irrelevant areas such as soft tissues, bone, vessels, fat, etc.

### 3.3. CNN and GRU-Based Lung Nodule Classification

Classification of lung nodules determined from CT images as benign or malignant is achieved using the DL model of CNN and GRU.

#### 3.3.1. Convolutional Neural Networks (CNN)

**Figure 5:**
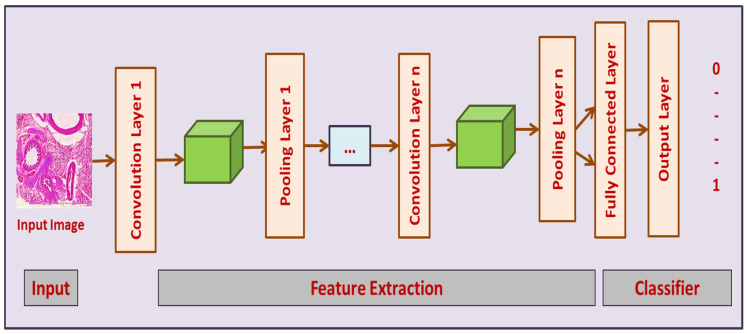
CNN architecture used for detecting infected lung nodules.

CNNs are involved in detecting image patterns. They have numerous front layers with which the network identifies lines and corners. The patterns can be transferred through a neural network (NN), and typical features can be identified by moving deep into the network (Khuriwal and Mishra 2018) [27]. This model is exceptionally effective for extracting image features. CNN is composed of 3 layers, which include pooling (P), convolutional (CL), and fully connected (FC) layers, as shown in Figure 5. CLs aid in determining neuron outcomes linked to local points. The dot product is determined for weights and regions. In input images, typical filters include pixels in small areas. These filters scan images by sliding a window on the image and controlling the recurring patterns which appear on the image area while scanning. Stride represents the distance amid filters in chains. Convolution can be extended to incorporate windows that overlap if the stride collection of parameters is not more than any filter dimensions.

#### 3.3.2. Gated Recurrent Unit Network (GRU)

**Figure 6:**
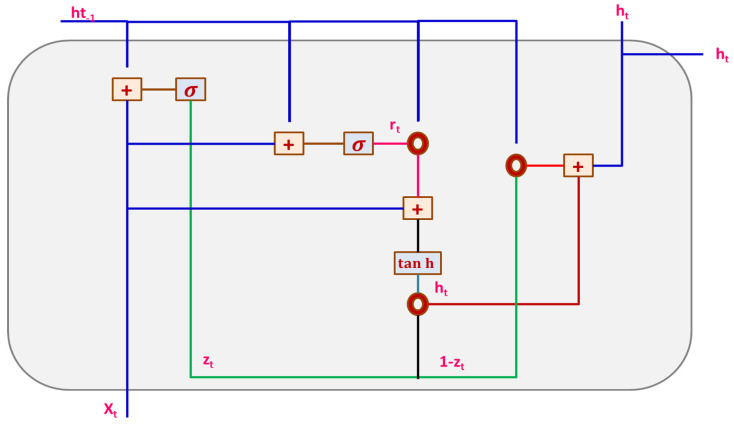
Structure of GRU Model.

The GRU network model is applied in a recurrent neural network (RNN) for handling the vanishing gradient issue. It is efficient compared to LSTM as it includes three main gates without the internal cell state. Information is concealed for safety in GRU, as shown in Figure 6. Forward and backward data is given to ‘Update gate (U)’. Moreover, details of former information are kept in ‘Reset gate (R)’.

The present memory gate uses ‘R’ for storing and maintaining the indispensable information present in the former state of the system. It is promising to include non-linearity in input by employing the ‘Input Modulation gate’ when concurrently offering features of zero mean. This is performed in a 2-fold way. Basic GRU gates are represented mathematically, as shown below.

(11) $$L_{t}=\sigma \left(U_{t}\cdot {\;K}_{X_{R}}+R_{t-1}W_{h_{R}}+C_{R}\right)$$

(12) $$M_{t}=\sigma \left(U_{t}\cdot {\;K}_{X_{U}}+R_{t-1}W_{h_{U}}+C_{U}\right)$$

where

$K_{X_{R}}$ and $K_{X_{U}}$—Weight factors with values of ‘$C_{R}$’ and ‘$C_{U}$’ biased.

#### 3.3.3. CNN-GRU

The CNN-GRU model includes 4 CLs, 3 max-pooling (MP), and 3 FC layers. Rectified linear units (ReLUs) are used to implement the activation function because neurons are not instantly activated, enabling the model to function better and enabling quick learning. Firstly, size images (50, 50, and 3) are given to CLs. The heights and widths of images are taken as 50 pixels with 3 channels each. This model demands features for performing feature extraction by moving through CLs1.

**Figure 7:**
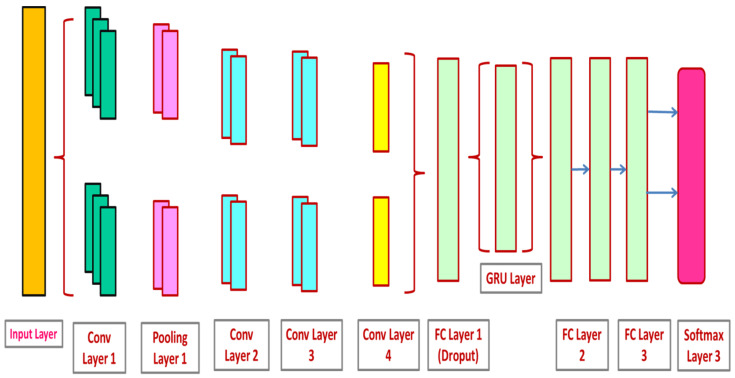
CNN-GRU architecture used for diagnosing lung cancer.

FM output is considered as 128 in this instance. Furthermore, the stride is set to 1 with a kernel size (3 × 3) in CLs1. ReLUs are used along with CLs1 to reduce the dimension of non-linearity. After primary CLs1, the output shape is 128 FMs, which is the size of (50, 50). Moreover, the MP layer reduces training parameters to (48, 48). The training parameter (48, 48, 128) is moved from the dropout layer following the MP layer to overcome overfitting-based issues. Primarily, the dropout of CL is set to 0.3. An added dropout of 0.9 is applied in the initial 2 FC layers to deal with overfitting. After every max pooling and CL, training factors intensely drop, succeeded by ReLUs and dropout. Once training is over, data is combined into a 1-D array and taken as input for implementing the FC layer. FM (512) along with training factor (32, 32) size is created through flattening. Once the complete process of 2D CLs is accomplished, dropout generates 256 FMs. The GRU model uses an FC layer with 512 neurons to handle the vanishing gradient challenge, after which 2 FLs are utilized. Figure 7 provides the CNN-GRU architecture used for diagnosing lung cancer. 

The SHOA-DNN mentioned above is used to classify CT images into benign or malignant.

## 4. Results and Discussion

The experimentation of the proposed SHOA-DNN model is conducted using the python 3.6.5 tool with the additional packages of OpenCV-python, pillow, sklearn, matplotlib, pickle, Numpy, Keras, and TensorFlow, which is GPU-CUDA enabled. This simulation process is achieved over the PC with the system configuration of 1 TB HDD, 250 GB SSD, 16 GB RAM, NVIDIA TITAN X, i5-8600k, and MSI Z370 A-Pro. The parameter setting for implementing the DNN model comprises an activation function (rectified linear unit (ReLU)), a drop rate of 0.25, a learning rate of 0.05, an epoch count of 15, and a batch size of 64, respectively. The experiment validation concerning the proposed SHOA-DNN model is attained using the benchmarked lung database LIDC/IDRI [25], which consists of 1018 CT images, and experienced thoracic radiologists reviewed these CT scans. The total number of images is classified into three class labels of standard, benign, and malignant. We take benign and malignant as positive cases, and the standard is negative.

**Figure 8:**
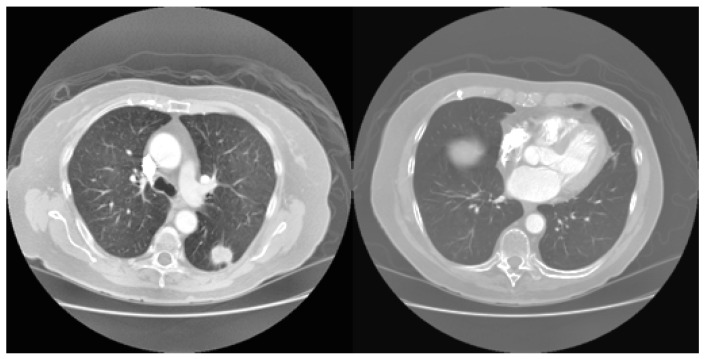
Sample CT images considered for experimenting with the SHOA-DNN model.

Some of the sample images considered for experimentation are presented in Figure 8.

### 4.1. Performance Evaluation Using Training and Testing Data with Distinct Classes

This performance evaluation of the proposed SHOA-DNN model was achieved with the training and testing ratio of 70:30. Table 1 presents the experimental results of the proposed SHOA-DNN model under the training and testing data ratio of 70:30. The results proved that the MCC, specificity, accuracy, precision, recall, and F-score achieved using the proposed SHOA-DNN model under the training and testing data ratio of 70:30 are 95.79%, 98.38%, 91.67%, 88.44%, 87.89%, and 87.93%, respectively [30,31].

**Figure 9:**
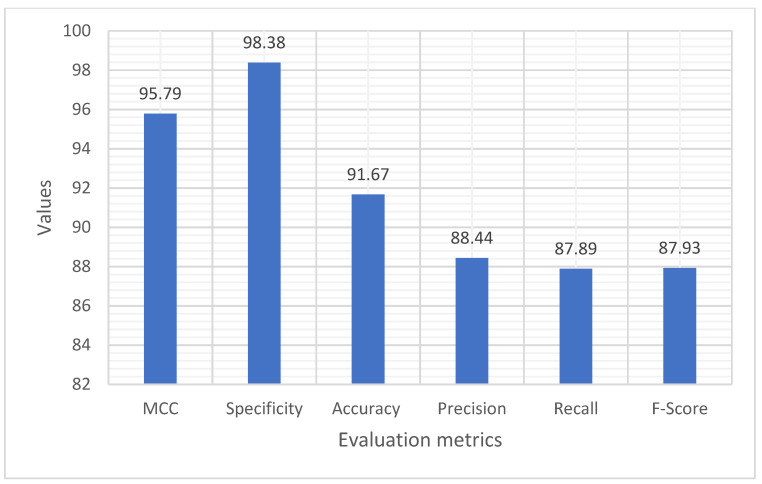
Results of the proposed SHOA-DNN model using average values under the 70:30 ratios of training and testing data.

Moreover, Figure 9 demonstrates the average value of the MCC, specificity, accuracy, precision, recall, and F-score achieved using the SHOA-DNN model under the training and testing ratio of 70:30. Independent of the training and testing data ratio, the proposed SHOA-DNN model performed well, as it incorporated the SHOA algorithm for optimizing the features that aid in a superior diagnosis model over the different classes of lung nodes considered for investigation.

### 4.2. Performance Assessment of Proposed SHOA-DNN Model and Compared Benchmarked Schemes

The performance of proposed and benchmarked schemes is analysed in terms of accuracy, precision, recall, specification, and F-score. Table 2 depicts the excellent performance of the proposed model over baseline schemes in terms of accuracy, precision, recall, specification, and F-score. From the outcomes, it is evident that the proposed SHOA-DNN model confirms better accuracy of 99.13%, which is improved by 4.21% compared to the benchmarked schemes for investigation. The proposed SHOA-DNN model facilitates this excellence in improved accuracy mainly due to two core reasons: (i) the adoption of SHOA aided in determining only the relevant characteristics for determining the exact features that contribute to better diagnosis; and (ii) the utilization of improved LBP during optimized feature extraction played an anchor role in the better classification process.

**Figure 10:**
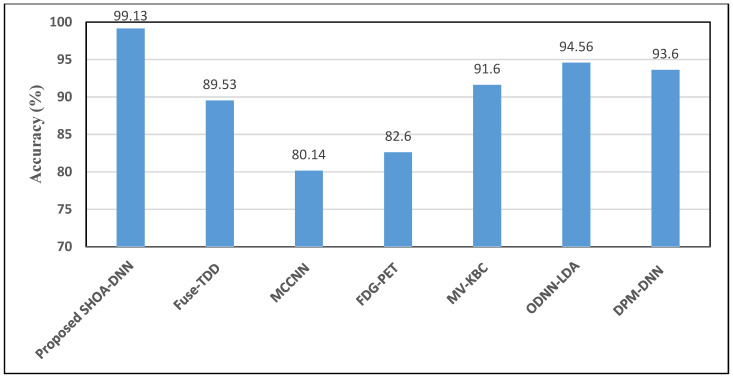
Comparative investigation of the proposed SHOA-DNN model using accuracy with the benchmarked schemes.

Figure 10 depicts the accuracy plots achieved using the proposed SHOA-DNN model and benchmarked mechanisms. The outcomes confirm that the proposed SHOA-DNN model, independent of the number of features, aided in a better classification process which helped categorize the CT images into benign and malignant lung nodules. The outcomes proved that the SHOA-DNN model improved accuracy by 21% better than the benchmarked schemes used for comparative investigation.

**Figure 11:**
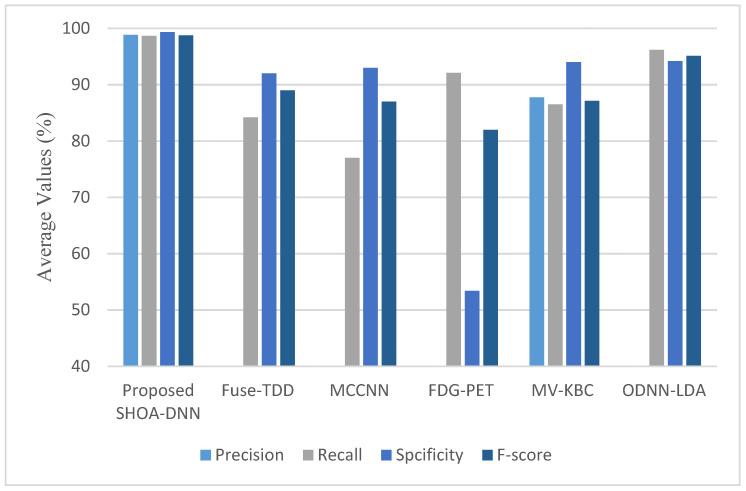
Results of the proposed SHOA-DNN model using average values under the 70:30 ratios of training and testing.

Further, Figure 11 presents the plots of precision, recall, specificity, and F-score achieved using the proposed SHOA-DNN model and standard schemes. Specifically, the precision results of the proposed SHOA-DNN model, on average, are improved by 5.82% over the benchmarked mechanisms. On the other hand, the recall value attained using the proposed SHOA-DNN model, on average, is improved by 4.96% over standard schemes. In addition, the specificity value confirmed via the proposed SHOA-DNN model, on average, is improved by 6.14% over standard schemes. In addition, the SHOA-DNN model improved the F-score by an average of 7.32% over the baseline approaches used for comparative investigation.

### 4.3. Performance Evaluation of the Proposed SHOA-DNN Using Training Time and Running Time

**Figure 12:**
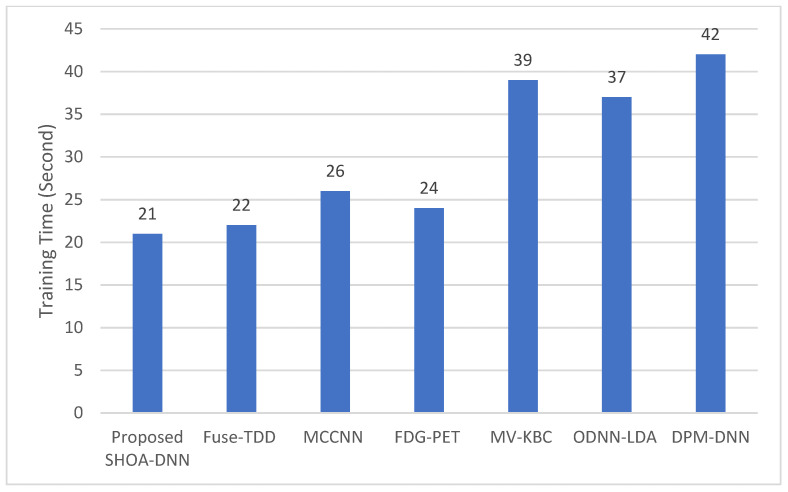
Results of the proposed SHOA-DNN model using average values under the 70:30 ratios of training and testing data.

This performance evaluation of the proposed SHOA-DNN model and benchmarked approaches are conducted using training time and running time. Figure 11 and Figure 12 present the plots of training time and running time incurred using the proposed SHOA-DNN model during the implementation process.

**Figure 13:**
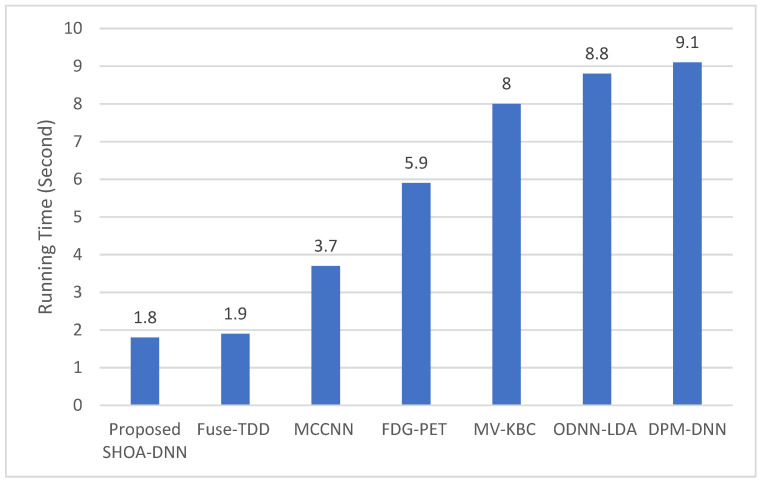
Results of the proposed SHOA-DNN model using average values under the 70:30 ratios of training and testing data.

The results confirmed that the proposed SHOA-DNN model reduced the running time, on average, by 6.79% from the benchmarked schemes. In addition, the training tome of the proposed SHOA-DNN model is also minimized, on average, by 8.92% in contrast with benchmarked schemes, as shown in Figure 13.

### 4.4. Performance Evaluation of the Proposed SHOA-DNN Using Cross-Validation

**Figure 14:**
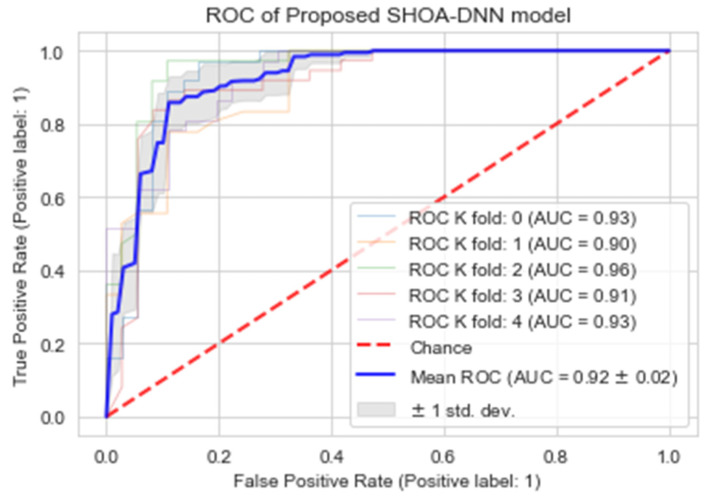
ROC of the proposed SHOA-DNN model using cross-validation (k = 5).

To evaluate the performance of the proposed SHOA-DNN model, we have deployed a cross-validation scheme (k-fold) to eliminate the factor of bias in the machine-learning model. We have selected the value of k = 5 in our experiment. The performance of the proposed model is assessed in terms of accuracy, recall, specificity, and MCC based on cross-validation. The results can be depicted in Table 3. Furthermore, we have also employed the receiver–operator characteristic curve (ROC) to validate the efficiency of the proposed model [32]. Figure 14 provides the performance of the proposed model based on the area under the curve (AUC) using cross-validation schemes.

## 5. Conclusions

The proposed SHOA-DNN model achieved increased accuracy during diagnosis of cancer lung nodules depending on the optimization of SHOA and classification of CNN-GRU. It adopts improved LBP and achieves potential image retrieval and determination of an optimized feature extraction process. It also used SHOA and confirmed the process of partitioning the nodules of cancer by defining the best characteristics, which aids in improving diagnostic accuracy. It utilizes an LBP to determine the appropriate feature retrieval from lung nodules. In addition, it adopts CNN and GRU-based classifiers for identifying whether the lung nodules are malignant or non-malignant depending on the features retrieved during the diagnosing process. The proposed work is compared with various classification methods, and the outcomes confirm that the proposed SHOA-DNN model reduced the running time, on average, by 6.79% over standard schemes. In addition, the training tome of the proposed SHOA-DNN model is also minimized on average by 8.92% from benchmarked schemes. As part of the future scope, it is also decided to develop an Orca predator optimization-based DNN model and compare its performance with the SHOA-DNN model under diversified characteristics of features utilized for the lung nodule cancer diagnosis process.

## Figures and Tables

**Table 1:** Results of SHOA-DNN model with training/testing-70:30.

Training/Testing-70:30						
	MCC	Specificity	Accuracy	Precision	Recall	F-Score
Normal	92.34	100.00	90.28	96.18	98.16	85.45
Malignant	96.15	97.88	93.56	88.29	90.58	89.42
Benign	98.78	98.15	91.19	100.00	74.94	86.71
Average	95.79	98.38	91.67	88.44	87.89	87.93

**Table 2:** Comparative results of the SHOA-DNN model with benchmarked schemes.

Compared Schemes	Accuracy	Precision	Recall	Specificity	F-Score
Proposed SHOA-DNN Model	99.13	98.84	98.64	99.32	98.72
Fuse-TDD [18]	89.53	-	84.19	92.02	89.00
MCCNN [20]	80.14	-	77.00	93.00	87.00
FDG-PET [22]	82.60	-	92.10	53.40	82.00
MV-KBC [23]	91.60	87.75	86.52	94.00	87.13
ODNN-LDA [24]	94.56	-	96.2	94.2	95.12
DPM-DNN [26]	93.60	-	-	-	-

**Table 3:** Results of Cross-validation analysis.

CV^1^	Accuracy (%)	Recall (%)	Specificity (%)	MCC (%)
K = 1	0.93	95.82	90.10	0.91
K = 2	0.90	98.35	88.56	0.87
K = 3	0.96	97.01	92.14	0.94
K = 4	0.91	89.25	100.00	0.88
K = 5	0.93	91.55	96.33	0.92
Mean	0.92	94.39	93.42	0.91

CV^1^ = Cross-validation.

## Data Availability

The data used to support the findings of this study are available from the corresponding author upon request.
